# Car seat impact on driver’s sitting behavior and perceived discomfort during prolonged real driving on varied road types

**DOI:** 10.1371/journal.pone.0259934

**Published:** 2021-11-16

**Authors:** Pascaline Lantoine, Mathieu Lecocq, Clément Bougard, Erick Dousset, Tanguy Marqueste, Christophe Bourdin, Jean-Marc Allègre, Laurent Bauvineau, Serge Mesure

**Affiliations:** 1 Aix-Marseille Univ, CNRS, ISM, Marseille, France; 2 Stellantis, Centre Technique de Vélizy, Vélizy-Villacoublay, France; National Tsing Hua University, TAIWAN

## Abstract

Prolonged driving under real conditions can entail discomfort linked to driving posture, seat design features, and road properties like whole-body vibrations (WBV). This study evaluated the effect of three different seats (S_1_ = soft; S_2_ = firm; S_3_ = soft with suspension system) on driver’s sitting behavior and perceived discomfort on different road types in real driving conditions. Twenty-one participants drove the same 195 km itinerary alternating highway, city, country, and mountain segments. Throughout the driving sessions, Contact Pressure (CP), Contact Surface (CS), Seat Pressure Distribution Percentage (SPD%) and Repositioning Movements (RM) were recorded via two pressure mats installed on seat cushion and backrest. Moreover every 20 minutes, participants rated their whole-body and local discomfort. While the same increase in whole-body discomfort with driving time was observed for all three seats, S_3_ limited local perceived discomfort, especially in buttocks, thighs, neck, and upper back. The pressure profiles of the three seats were similar for CP, CS and RM on the backrest but differed on the seat cushion. The soft seats (S_1_ & S_3_) showed better pressure distribution, with lower SPD% than the firm seat (S_2_). All three showed highest CP and CS under the thighs. Road type also affected both CP and CS of all three seats, with significant differences appearing between early city, highway and country segments. In the light of these results, automotive manufacturers could enhance seat design for reduced driver discomfort by combining a soft seat cushion to reduce pressure peaks, a firm backrest to support the trunk, and a suspension system to minimize vibrations.

## Introduction

Transportation habits are evolving, with people driving more often and farther every year [1]. The French, for example, currently make an average of three car trips a day covering about 60 kilometres over 90 minutes [2]. The major risk is that prolonged driving exposes drivers to discomfort or fatigue [3–6]. The feeling of discomfort refers to physical symptoms and predicts the occurrence of pain, in particular in the back, neck, buttocks and thighs [7, 8]. Thus, one of the most important purchasing criteria for drivers is comfort, which is why an important design criterion for car manufacturers is minimizing discomfort, particularly on the seat.

Ebe & Griffin [9] determined two main types of factors, “static” and “dynamic”, which may influence perceived driver discomfort. Static factors include seat design characteristics such as seat shape, dimensions, or materials. These define the driving posture itself, with muscular, biomechanical, and vascular consequences. Sitting causes trunk flexion, which increases muscle stiffness as well as the mechanical load applied on the spine and the compression of tissues under buttocks, thighs, and back [10, 11]. In addition, driving is associated with postural fixity and isometric muscle contractions induced by the need to reach commands and pedals [12, 13]. Dynamic factors refer mainly to vibrations through the seat, which are directly dependent on the vehicle and road properties. In real conditions, the quality of the road is random and associated whole-body vibrations (WBV) can affect driver’s posture, discomfort, and driving performance [14, 15]. The magnitude of lateral and longitudinal accelerations perceived by the driver depends on the road’s linearity, the quality of the vehicle’s suspension system, the speed, and the roughness of the road surface [16]. Mansfield et al. [17] further developed a multi-factorial model of vehicle seat discomfort incorporating these “static” and “dynamic” factors while adding the temporal effect of driving. The authors found that, as the magnitude of WBV or time increased, so did the feeling of perceived discomfort.

Because maintaining a driving posture for an extended period necessarily generates discomfort, drivers may try to reduce this discomfort or its frequency by moving on their seat, despite the restricted cockpit space. According to Baucher & Leborgne [10], the seat should encourage mobilization of trunk and limbs to relax muscles and relieve soft tissue and intervertebral disk compressions. Several authors reported an increase in repositioning movements (RM) with driving time, linked to changes in perceived discomfort [18–20]. Vergara & Page [21] identified two types of RM: macro-movements, referring to large postural changes and micro-movements, referring to oscillations around a “stable” posture. They found macro-movements to be the result of perceived discomfort. Of the various methods used to determine RM, pressure maps are among the most common, allowing sitting strategies at the seat-driver interface to be identified [22, 23]. Makhsous et al. [24] evaluated the influence of seat characteristics, particularly foam stiffness, on pressure parameters, using a soft cushion to induce a larger contact surface, for example. The composition of the foam appeared to be a major design criterion, affecting pressure distribution and the body’s maintenance in an upright posture, and thus influenced feelings of discomfort. Moreover, pressure parameters depend on driving conditions. Seat cushion contact over time was reported to lead to sitting changes observable through a loss of pressure homogeneity and stabilization of contact pressure and surface as time increased [25–27].

For these reasons, seat designers need to take into account specific types of interaction between seat, driver, and road environment to optimize static and dynamic seat factors, in order to reduce discomfort and its related harmful physical consequences on drivers [28–30]. In the agricultural vehicle and truck contexts, one way to minimize transmission of vibrations in the vertical axis (*z*-axis) is to supplement the seat with a suspension system [30, 31]. This innovation can be used in a car to attenuate WBV and delay the onset of perceived discomfort, in particular in the back, by improving seat dynamic properties [17]. However, there is insufficient data on its performance under real driving conditions that induce varying WBV and different lateral and longitudinal solicitations. Moreover, the benefit from seat suspension systems in cars for personal use remains to be explored.

This study analyzed the evolution of the driver’s sitting behavior by measuring pressure distribution and perceived discomfort under real prolonged driving conditions. Using real conditions enabled us to consider the effect of random road roughness due to surface asperity, which may induce vibrations varying in magnitude in *z*-axis. It also allowed us to take into account the effect of varying road types involving different levels of acceleration. Under these conditions, we investigated how (1) different seats (static factor) and (2) real driving (dynamic factor) affected pressure parameters (RM, pressure distribution, contact surface, and contact pressure) and subjective general and local discomfort. Three seats with different properties were tested: one soft (S_1_), one firm (S_2_), and one soft with a suspension system (S_3_), on different road types. Based on previous research on agricultural vehicles we expected S_3_, with its better dynamic properties, to improve the prolonged driving experience by reducing perceived discomfort.

## Materials & methods

The study was approved by automotive manufacturer Stellantis’ “Comité d’hygiène, de sécurité et des conditions de travail” (Hygiene, Safety and Working Conditions Committee) and the research site was validated as biomedical research site n˚15–225 by the “Agence Régionale de Santé - Ile de France” (regional health agency Ile de France).

### Participants

Twenty-one employees from Stellantis, a leading automotive manufacturer, were recruited through an email campaign and volunteered to participate in the experiment (16 men & 5 women; height: 1.74 ± 0.07 m; weight: 74.05 ± 13.75 kg). No participant had previous experience of a driving protocol, and all were naïve to the aim of comparing hardness of seats and road types in this experiment. The inclusion criteria were holding a driving license for a minimum of two years. Participants were excluded if they had suffered back pain or musculoskeletal disorders in the previous year, or if such symptoms had required medical follow-up and/or time off work. Subjects who had previously undergone back surgery were also excluded. Before each experimental session, participants completed a questionnaire about their driving habits (driving licence tenure: 25.1 ± 10.0 years; annual mileage: 13 650 ± 8777 km; daily driving time: 78 ± 75.5 min) and gave their written informed consent (as outlined in PLOS consent form*)*. Participants could stop the experiment at will and took responsibility for any violation of the Highway Code. Moreover, they were informed that experimenters would answer their questions about the aim of the study at the end of the driving sessions.

### Experimental car

For this experiment, a 3008 vehicle (puretech 130, Peugeot, France) with an automatic gearbox was used. As a consequence, there was no clutch pedal and no gear switching. Technical adaptations were made to the car to allow each seat to be installed in the driver’s place, and to collect data. Additional substructures were designed to fix seats to the car floor and to maintain the original cockpit configuration of the standard 3008 seat. Several electronic devices, in particular two cameras, one facing the driver and one oriented towards the road, a power station, and a computer, were installed in the car cockpit and trunk. The computer screen was fixed to the back of the front passenger seat facing the second experimenter, who used it to collect pressure and video data.

### Experimental synopsis

**Fig 1:**
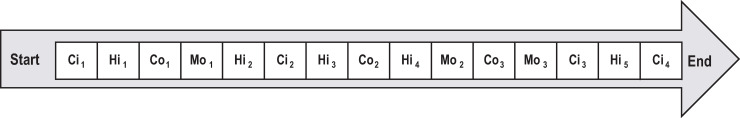
Chronogical order of road segments. Participants drove on 15 segments of 4 road types: 4 City segments (Ci), 5 Highway segments (Hi), 3 Country segments (Co), and 3 Mountain segments (Mo).

All driving sessions covered the same 195 km itinerary lasting a minimum of 3 hours in real driving conditions. Driving sessions were scheduled at least one week apart and involved participants randomly assessing one seat among the three tested. Each experimental session started from the Technical Center of French car manufacturer PSA (Vélizy-Villacoublay, France) at the same time of the day (8:00 am) to avoid any effect from differences in circadian rhythms and traffic between sessions. Upon the participant’s arrival, pressure mats were installed in the middle of the backrest and cushion. Then, participants were asked to put on a sports outfit provided by the experimenters, to avoid any effect from clothing type on perceived discomfort and any seam marking on pressure mats. They got into the cockpit and adjusted the seat to be as comfortable as possible. The participants could adjust the fore-aft position of the seat, the backrest inclination, the height of the seat, and the height of the steering wheel. Once adjusted, these settings subsequently remained unchanged throughout the driving session. Before the driving session, a calibration of two minutes in referential position (right foot on the throttle pedal, left foot on rest platform, left knee at an angle of 90°, and hands on the steering wheel at positions nine and three) was recorded by pressure system XSENSOR^®^ and by two cameras. This calibration was defined as the baseline posture for the future normalization of pressure data representative of posture in an automatic car. After the calibration period, the participant started the vehicle and drove to the starting point. Then the two experimenters present throughout the driving session, one in the front passenger seat monitoring perceived discomfort measures and one at the rear handling recordings, gave the last instructions and launched pressure software and cameras as the driving task began. They explained that the vehicle was an automatic car and that consequently only the right foot could be used to activate gas and brake pedals. Moreover, drivers were allowed to move in their seat during the driving session if they felt uncomfortable, while still complying with the experimenter’s instructions. The itinerary was displayed on a cellphone GPS and was composed of four types of road segments: city roads (Ci), country roads (Co), highways (Hi) and mountain roads (Mo). Each road type was repeated three times at least during the itinerary, yielding 15 road segments analyzed in the chronological order presented in Fig 1. Drivers had to respect French driving rules, which included keeping their hands on the steering wheel and not exceeding speed limits. After the entire itinerary had been followed to the finishing point, data recording ended.

### Seats tested

**Fig 2:**
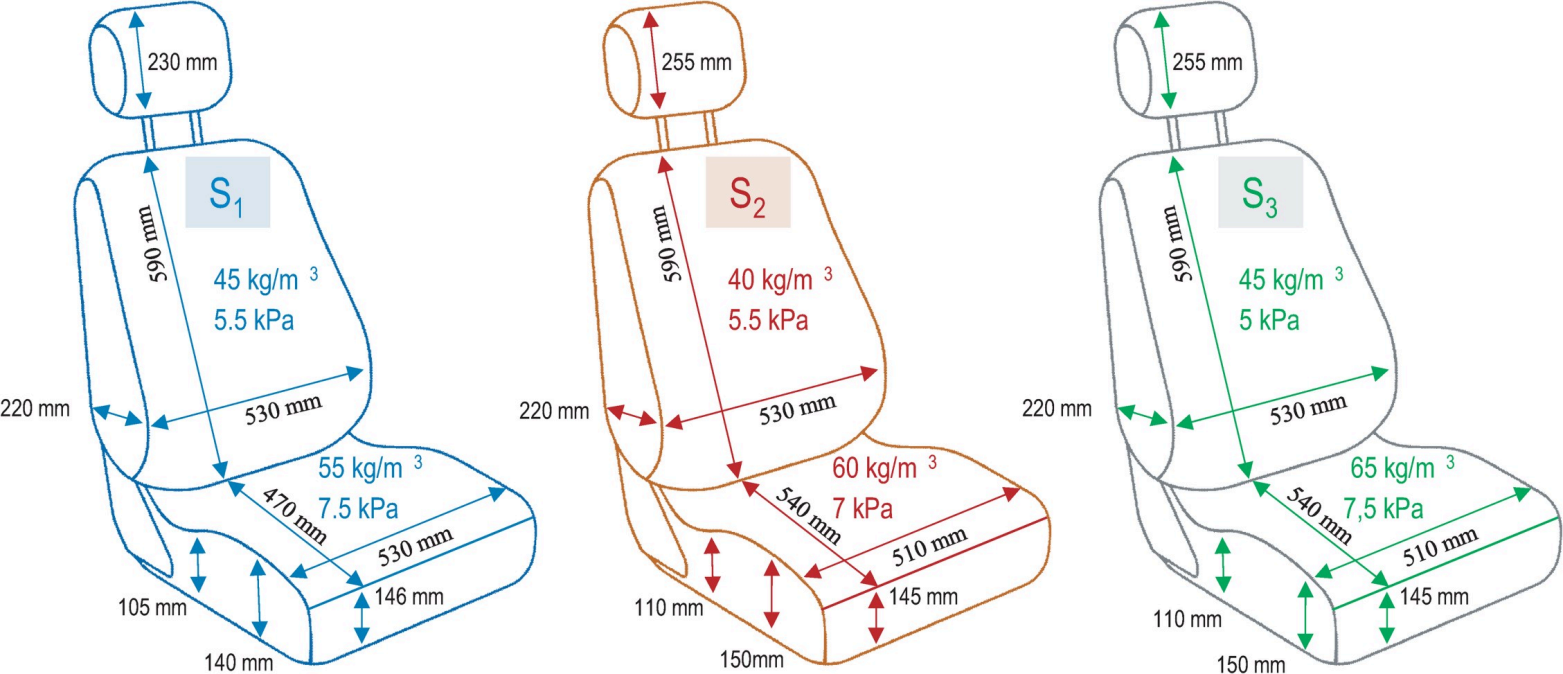
Design details of the seats. For each seat, main geometrical dimensions and values of bearing capacity and density for the seat cushion and backrest.

**Fig 3:**
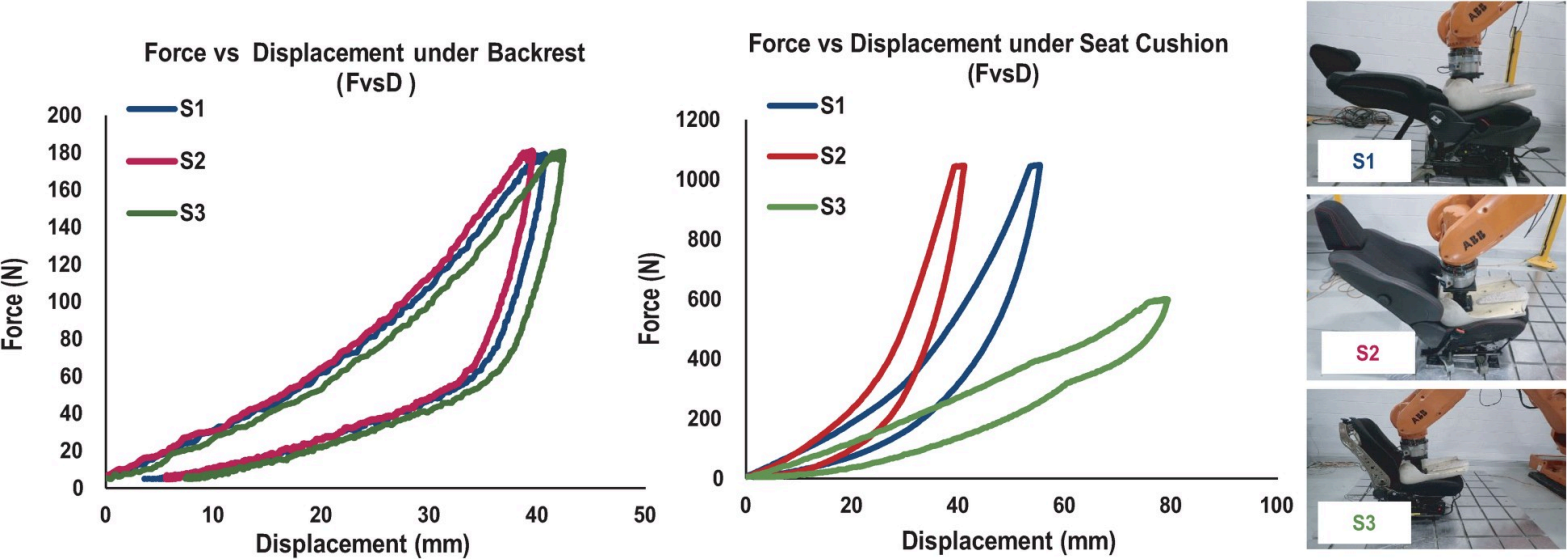
Force vs Displacement (FvsD) for the three seats tested. A test of foam hardness was conducted at CTAG and is illustrated in the pictures. The graph illustrates the FvsD for each seat cushion and each backrest. The force was applied by a rigid dummy. For the same force applied, cushion displacement was highest for S_3_, followed by S_1_ and finally S_2_, thereby defining S_3_ and S_1_ as soft seats and S_2_ as a firm seat.

Three different seats were tested, two already commercially available and the third a prototype based on a new technology. The three seats had the same metal frame and the same mechanical adjustments, but different covers, foam densities, and absorption properties. The first seat (S_1_) is a Citroën seat considered soft, covered in a classic upholstery fabric. The second (S_2_) is a firmer Peugeot seat with mixed fabric and leather upholstery. The third (S_3_) is the softest seat and a prototype using a suspension-enhancement technology described below. Details of dimensions and foam densities are given in Fig 2. In addition, softness was defined according to a standard test of Force vs Displacement (FvsD) conducted at CTAG (Automotive Technology Center of Galicia, Spain), which records the maximum displacements of foam under force. FvsD under the backrest was similar for the three seats but the seat cushions’ FvsD differed. All FvsD results are shown in Fig 3. S_3_ incorporated a vibration absorber behind the backrest, a design innovation that allows better absorption of vibrations in z-axis, using hinged bars to enhance the car’s suspension. The aim is to reduce the resonance frequency experienced by the driver, which is between 4 and 8 Hz and can be a source of discomfort [32].

### Measurements

#### Pressure and repositioning movements

Pressure parameters and RM were analysed via the pressure system XSENSOR^®^ Technology Corporation (Xsensor, Inc., Calgary, Alberta, Canada). The system consisted of two sensor pressure mats (model X3 LX100) placed on the backrest (40 x 64 sensors, 50.8 cm x 81.2 cm sensing area, mat LX 100:40.64.02) and on the cushion (40 x 40 sensors, 50.8 cm x 50.8 cm sensing area, mat LX 100:40.40.02). Using the associated software (XSENSOR Pro V8), acquisition frequency was set at 1 Hz to ensure detection of postural changes, whose frequency range is known to be lower. This system afforded a measurement range of between 0.07 and 2.7 N/cm^2^ and a measurement threshold of 0.07 N/cm^2^. Moreover, it provided an accuracy of ± 10 mmHg and uncertainty was calculated at ± 5.9 mmHg.

The two webcams installed, one facing the driver and one facing the road, each had a sampling frequency of 30 frames per second. The aim of the video recording was to detect repositioning movements due to feelings of discomfort as distinguished from those simply inherent to the driving task.

#### Perceived discomfort

Throughout the driving sessions, participants were asked to evaluate their perceived discomfort, assessing it every twenty minutes from the beginning (t_start_ at 3 min) to the end (t_end_ at 183 min) of the itinerary. A total of ten assessments were collected on two different types of discomfort. The first, whole-body perceived discomfort, was evaluated using a Visual Analogue Scale (VAS). One side of the VAS was composed of a slider that drivers could move to score their whole-body discomfort, while the other side was graduated to allow the experimenter to numerically quantify the driver’s discomfort score. The second evaluation, of local discomfort, required participants to verbally report their feeling of discomfort for each body area separately (neck, upper-back, lower-back, arms, buttocks, thighs, legs, and feet). For reasons of organization and security, these verbal assessments of local perceived discomfort were recorded and scored from 0 (no discomfort) to 100 (maximum level of discomfort imaginable). Local perceived discomfort was expressed as a percentage of the maximum value given by each participant during each driving session.

### Data processing

#### Pressure analysis

All pressure data were analyzed via a MATLAB program (vR2017a, MathWorks Inc., Natick, USA), which calculated the contact pressure (CP) and the contact surface (CS) for both pressure mats. The seat pressure distribution percentage (SPD%), representing the homogeneity of pressure distribution [33], was calculated only for the cushion mat. The lower the SPD%, the more uniform and homogeneous the pressure distribution. The first step of the analysis consisted in importing the calibration into the program so as to include the driver’s weight and contours applied at the interface with each seat.

**Fig 4:**
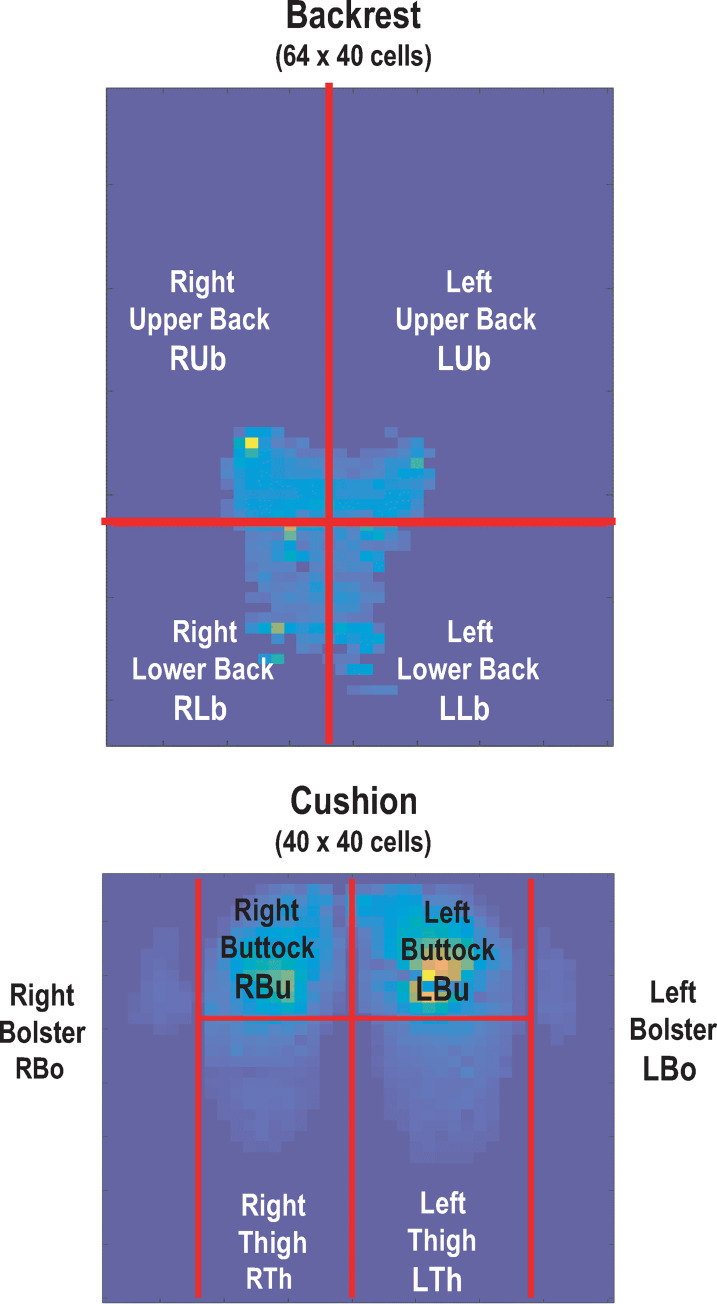
Representation of pressure mat areas. Spatial division of pressure mats to analyze 4 areas for the backrest (Right/Left Upper back and Lower back) and 6 areas for the cushion (Right/Left Buttocks, Thighs and Bolsters).

After calculation of CP and CS for the full mats, spatial cutting was performed to divide each mat in such a way as to obtain CP and CS for six cushion areas: [Right/Left Bolsters (RBo/LBo), Right/Left Buttocks (RBu/LBu) and Right/Left Thighs (RTh/LTh)] and for four backrest areas [Right/Left Upper back (RUb/LUb) and Right/Left Lowerback (RLb/LLb)] (Fig 4).

A temporal division of all pressure parameters according to time segments was performed for all subjects and all seats. Moreover, all pressure parameters were normalized in terms of time spent in each road segment according to areas under the curve for each pressure parameter. This was calculated as follows: AUC of segment n / time spent in segment n (sec). However, pressure parameters were not normalized in terms of driver’s bodyweight due to our within-subjects design. Pressure parameters were expressed as mean ± standard error of the mean (SEM) for each normalized road segment.

#### Specifications for repositioning movements

A MatLab program was used to dynamically read backrest pressure and surface data and to calculate changes in trunk posture. Only macro-movements were considered as repositioning movements. Prior to RM identification, a calculation of the force applied (F) on the backrest was performed, combining CP and CS data. Then, a sliding one-minute average was continuously calculated all along the signal of F. A comparison between the F-value of each frame at *t*-moment and the corresponding moving-average at the same *t*-moment was used to determine whether the frame was an RM. If the value of F at this *t-*moment exceeded 3 standard deviations (STD) from the moving-average, then the frame was considered as an RM. If the value was below 3 STD from the moving-average, the RM was defined as a *forward repositioning movement* of the trunk (FRM) because the F value was suddenly lower than the moving-average. Conversely, if the value was above 3 STD from the moving-average, the RM was defined as a *backward repositioning movement* of the trunk (BRM) because the F value was suddenly higher than the moving-average. RM frequency per minute was used to analyze FRM and BRM.

### Statistical analysis

The normality of all data was tested and validated with a Shapiro-Wilk test. Then, the homogeneity of variance was verified with a Levene test. For all statistical tests, one categorical independent variable (3 seats) was used. To analyze contact pressure (CP) and contact surface (CS) for both backrest and cushion, and SPD% for cushion alone, a one-way ANOVA (15 segments) was performed. Further data on evolution of CP and CS for each area of backrest and cushion were obtained through additional repeated measures ANOVA (15 segments x 6 cushion areas; 15 segments x 4 backrest areas), and the frequency of RM was also analyzed using repeated measures ANOVA (15 segments x 2 types of RM).

In addition, to analyze discomfort scores, a one-way ANOVA (10 discomfort scores) was performed both on whole-body scores and on scores for each body part. Newman-Keuls post-hoc tests were performed if a significant main effect was found. Differences were considered significant at p<0.05. All statistical analyses were conducted with Statistica (v13, TIBCO Software Inc., USA). All data are reported as mean ± SEM.

## Results

### Cushion

#### Full cushion mat

**Fig 5:**
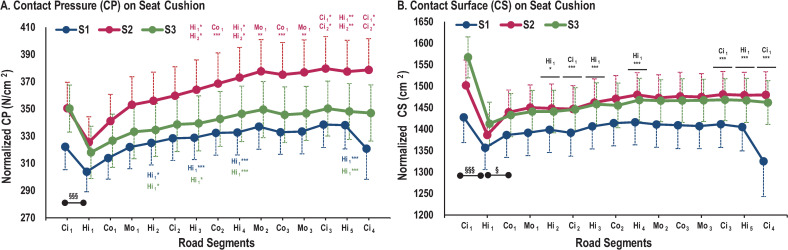
Pressure parameter evolution on full cushion mat for each road segment. A) Evolution of CP. B) Evolution of CS. * represents significant differences between segments for the same road type (*: p<0.05; **: p<0.01; ***: p<0.001). Colored * represents one seat, black * represents three seats. § represents significant differences between two successive segments (§: p<0.05; §§: p<0.01; §§§: p<0.001).

For CP, a SEATxSEGMENT interaction effect was observed (*F*_(28,560)_ = 1.84; *p*<0.01). For all three seats, there was no statistical difference between any two successive road segments, except between Ci_1_ and Hi_1_ (Fig 5A). The post-hoc test revealed a lower CP in Hi_1_ compared to other highway segments for S_1_ and S_3_ (*p*<0.05). For S_2_, CP was lower in the first six (Ci_1_, Hi_1_, Co_1_, Mo_1_, Hi_2_ and Ci_2_) than in the later segments of a given road type (*p*<0.05).

**Fig 6:**
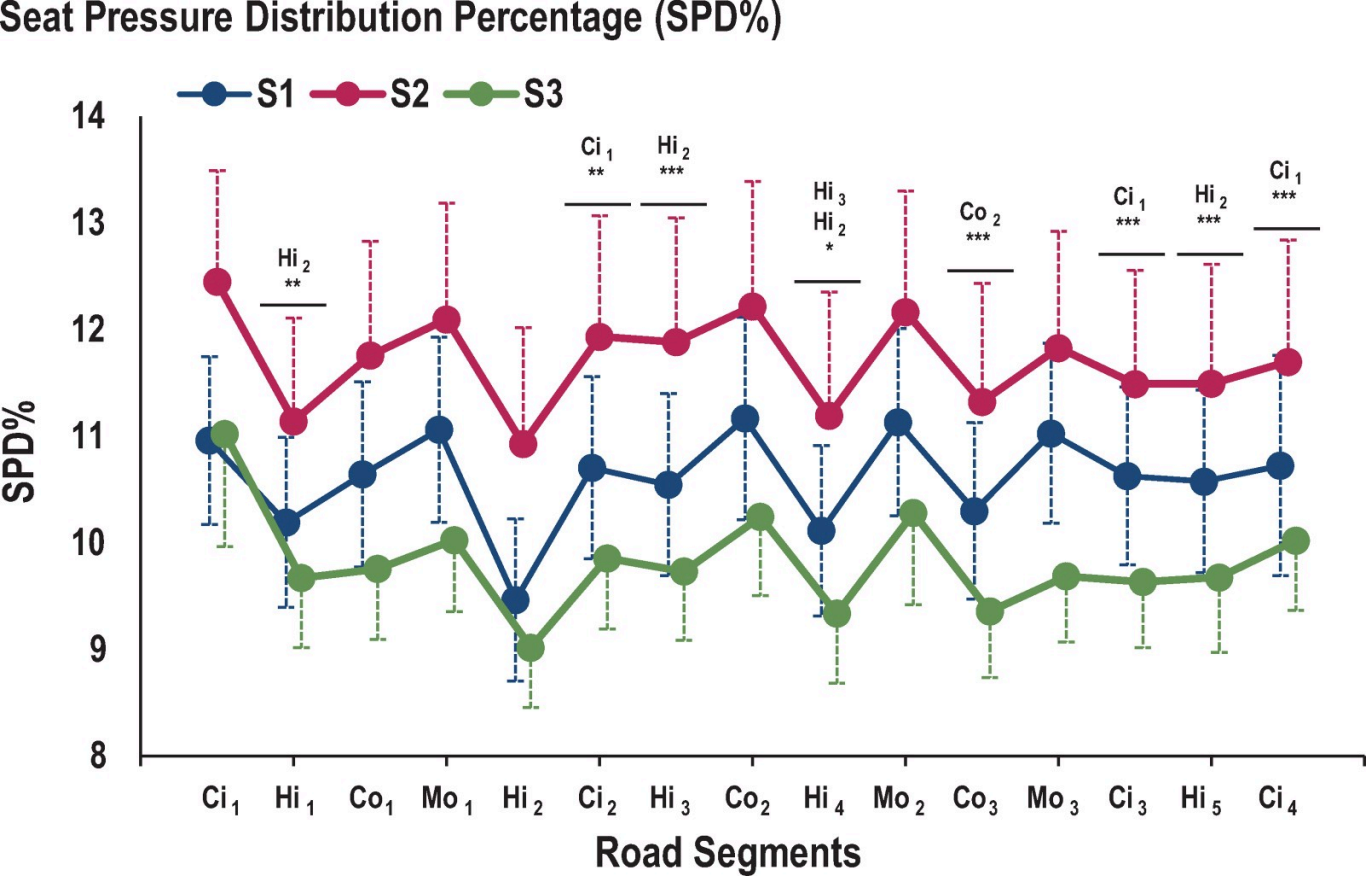
Evolution of SPD%. Evolution of the uniformity or pressure distribution represents with SPD% score. * represents significant differences between segments for the same road type (*: p<0.05; **: p<0.01; ***: p<0.001).

Regarding CS and SPD%, a main SEGMENT effect was observed (*F*_(14,560)_ = 6.55; *p*<0.001 and *F*_(14,560)_ = 13.00; *p*<0.001, respectively) (Fig 5B). Whatever the seat, post-hoc testing revealed significant differences between the three first successive segments with higher CS in Ci1 than in Hi1 and lower CS in Hi1 than in Co1 (*p*<0.05). Whatever the seat, post-hoc tests revealed higher CS in Ci_1_ than in all subsequent city segments (*p*<0.01) and lower CS in Hi_1_ than in all subsequent highway segments (*p*<0.01). Post-hoc tests showed a higher SPD% in Ci_1_ than in subsequent city segments (*p*<0.01), a higher SPD% in Co_2_ than in Co_3_ (*p*<0.001), and a higher SPD% in Hi_3_ than in Hi_4_ (*p*<0.05). In contrast, SPD% was lower in Hi_2_ than in all other highway segments (*p*<0.05) (Fig 6).

#### Areas of cushion mat

**Fig 7:**
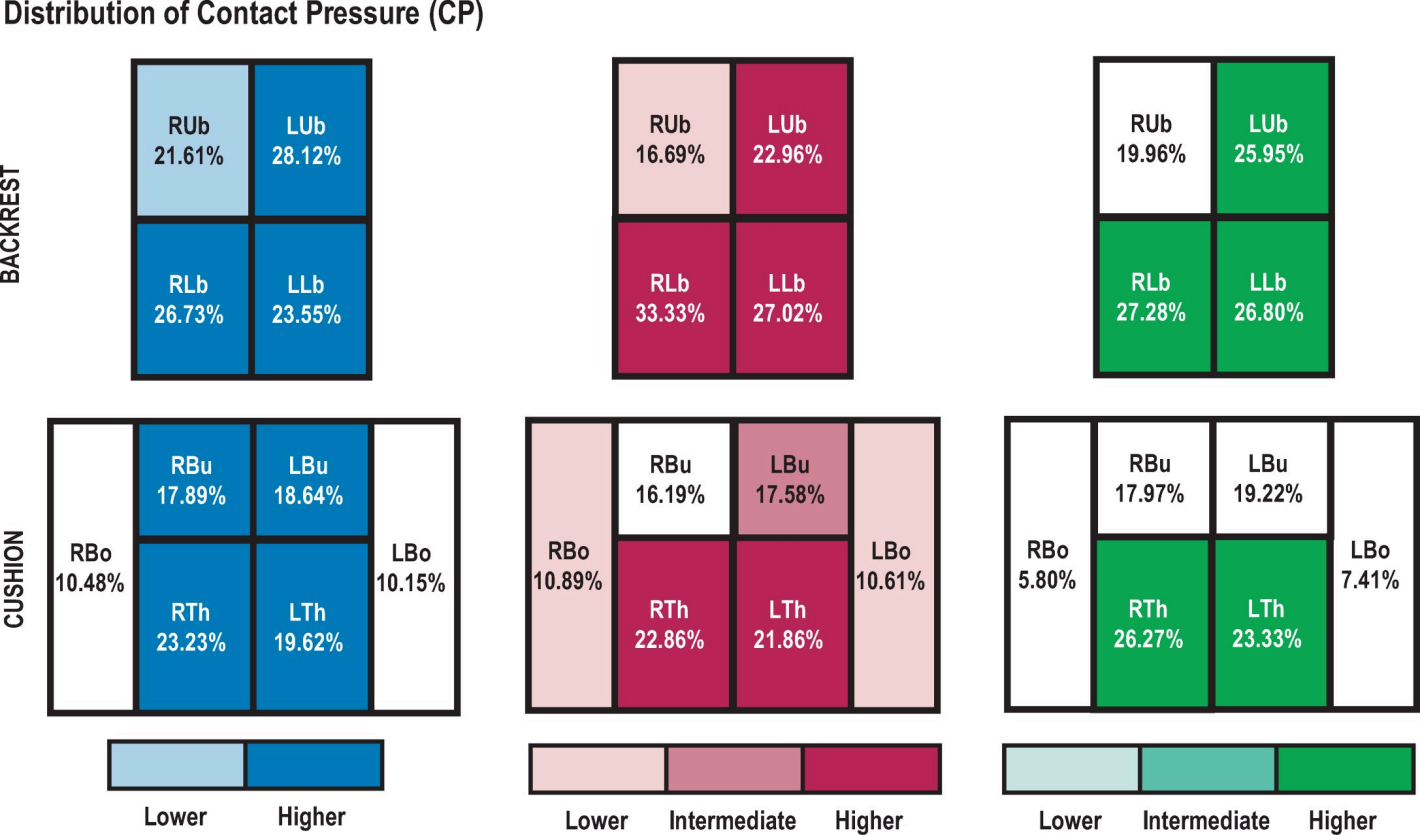
CP profiles. Distribution of CP by area of backrest and cushion. Each shade of color represents a significant difference (p<0.05).

For CP, an AREAxSEAT interaction effect was found (*F*_(10,200)_ = 2.52; *p*<0.01). Whatever the road segment, post-hoc tests revealed that both bolsters (RBo and LBo) of S_1_ had a lower CP than the other seat cushion areas (*p*<0.001). For S_2_ and S_3_, CP in RBo and LBo were lower than in both thighs (RTh and LTh) and buttocks (RBu and LBu) (*p*<0.01 with S_2_; *p*<0.001 with S_3_). Moreover, both seats showed higher CP in RTh and LTh than in RBu and LBu (*p*<0.05; see Fig 7 for detailed breakdown of CP and statistical differences for the three seats).

In addition, an AREAxSEGMENT interaction effect (*F*_(70,2800)_ = 6.99; *p*<0.001) was also observed on CP. Post-hoc tests indicated that whatever the seat, CP for all seat areas was significantly lower in Hi_1_ and Co_1_ than in all subsequent highway segments (Hi_2_, Hi_3_, Hi_4_, Hi_5_) and country segments (Co_2_, Co_3_) (*p*<0.001). In highway segments, CP for RBu and RTh increased with time, showing significantly higher values with successive highway segments (Hi_1_ < Hi_2_ < Hi_3_ < Hi_4_ < Hi_5_; *p*<0.001). Regarding the four other areas of the seat cushion (LBu, LTh, RBo, LBo), CP was lower in Hi_2_ than in Hi_3_, Hi_4,_ and Hi_5_ (*p*<0.001). Except for RBo and RBu, all seat cushion areas showed lower CP in Mo_1_ than in the subsequent mountain segments (Mo_2_ and Mo_3_; *p*<0.001). CP values in Ci_1_ were significantly lower than in all subsequent city segments (Ci_2_, Ci_3_, Ci_4_) for RBo (*p*<0.001) and lower than in Ci_3_ for RTh (*p*<0.001). In Ci_2_, CP values were also lower than in Ci_3_ for LBu, Rbo, and LBo (*p*<0.001).

**Fig 8:**
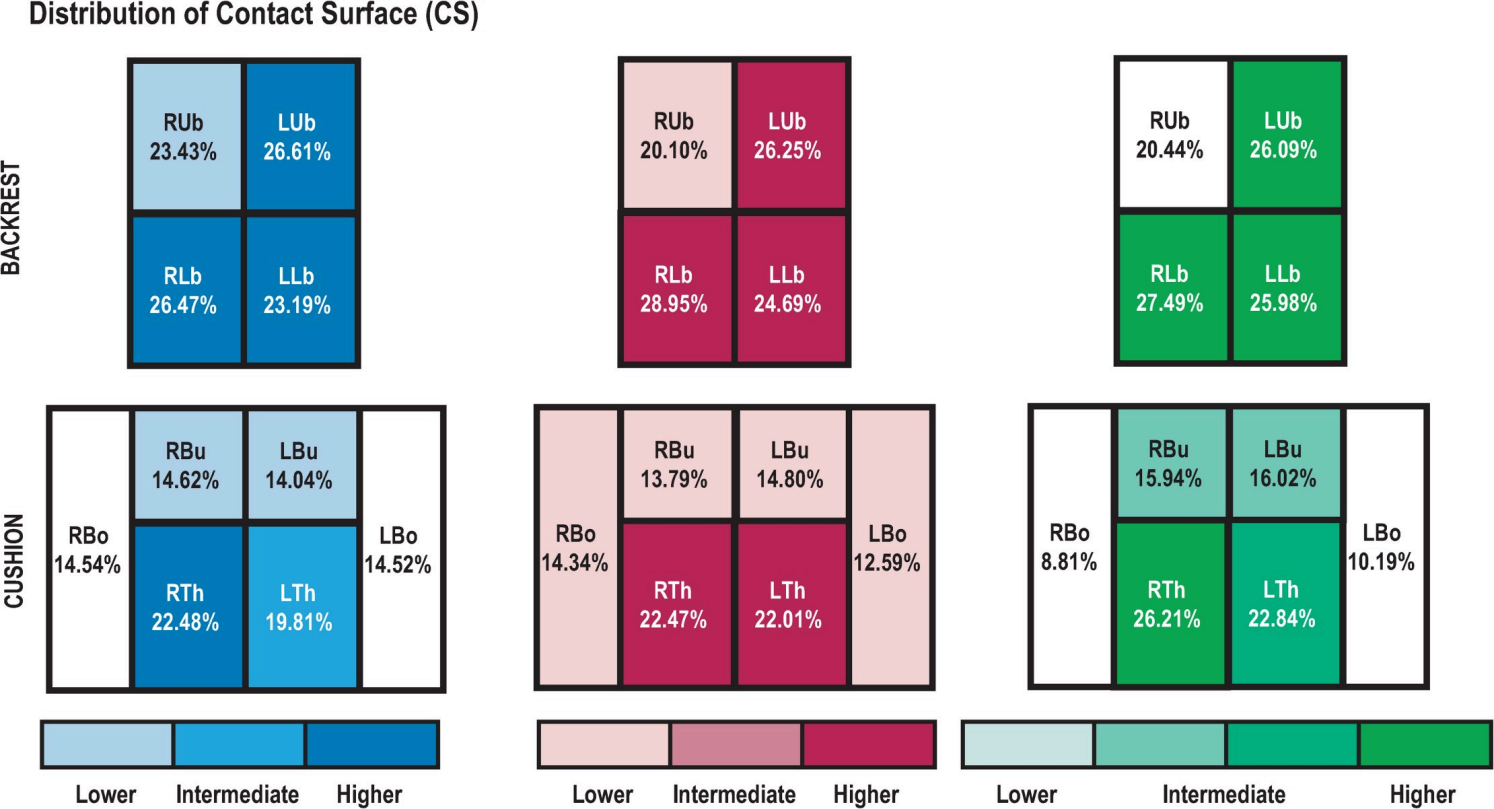
CS profiles. Distribution of CS by area of backrest and cushion. Each shade of color represents a significant difference (p<0.05).

Regarding CS, seat cushion measurements indicated an AREAxSEAT interaction effect (*F*_(10,200)_ = 5.75; *p*<0.001). Whatever the road segment, post-hoc tests showed lower CS in RBo for S_3_ than for S_1_ and S_2_ (*p*<0.01). In addition, S_3_ had higher CS in RTh than S_1_ and S_2_ (*p*<0.05), but lower CS in LTh than S_1_ (*p*<0.05). With S_1_, CS was higher in RTh than in all other areas (*p*<0.05), and was also significantly higher in LTh than in RBu, LBu, RBo and LBo (*p*<0.01). S_2_ showed significantly higher CS in RTh and LTh than in other areas. S_3_ showed lower CS in RBo and LBo than in thighs and buttocks (*p*<0.001), significantly lower CS in RBu and LBu than in thighs (*p*<0.01), and significantly higher CS in RTh than in LTh (*p*<0.001; see Fig 8 for details of CS distribution and significant differences for the three seats).

CS values were also influenced by an AREAxSEGMENT interaction effect (*F*_(70,2800)_ = 12.80; *p*<0.001). Post-hoc tests showed that whatever the seat, CS for all seat areas except bolsters was significantly higher in Ci_1_ than in all subsequent city segments (Ci_2_, Ci_3_, Ci_4_, *p*<0.001). In Hi_1_, CS in bolsters and thighs was lower than in Hi_3_, Hi_4,_ and Hi_5_ (p<0.05), and significantly lower in Hi_2_ than in subsequent highway segments (Hi_3_, Hi_4,_ and Hi_5_; *p*<0.01). In Co_1_, CS for RBo alone was significantly lower than in Co_2_ and Co_3_ (*p*<0.001). In Mo_1_, CS for LBo was lower than in other mountain segments (Mo_2_ and Mo_3_, *p*<0.01).

### Backrest

#### Full backrest mat

**Fig 9:**
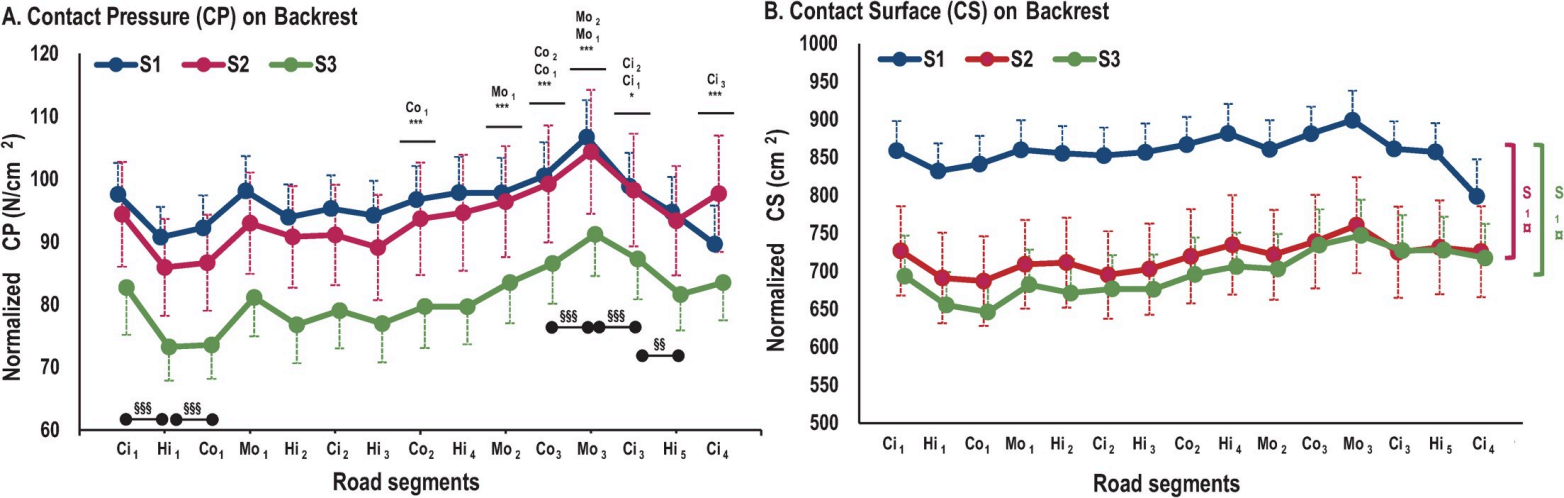
Pressure parameter evolution on full backrest mat for each road segment. A) Evolution of CP. B) Evolution of CS. Symbol * represents significant differences between segments for the same road type (*: p<0.05; **: p<0.01; ***: p<0.001). Colored * represents one seat, black * represents three seats. § represents significant differences between two successive segments (§: p<0.05; §§: p<0.01; §§§: p<0.001). ¤ represents significant differences between seats (¤: p<0.05; ¤¤: p<0.01; ¤¤¤: p<0.001).

A main SEGMENT effect was observed for CP (*F*_(14,560)_ = 22.22; *p*<0.001) (Fig 9A). Whatever the seat, CP was higher in Ci_3_ than in Ci_1_ (*p*<0.05) and Ci_2_ (*p*<0.001) but lower than in Ci_4_ (*p*<0.001). CP was significantly lower in Hi_1_ than in all subsequent highway segments (*p*<0.05). For country and mountain segments, CP significantly increased with time. CP was lower in Co_1_ than in Co_2_ (*p*<0.001) and these two first country segments had lower CP than Co_3_ (*p*<0.001). CP was lower in Mo_1_ than in Mo_2_ (*p*<0.001) and these two first mountain segments had lower CP than Mo_3_ (*p*<0.001).

Backrest measurements of CS indicated a SEATxSEGMENT interaction effect (*F*_(28,560)_ = 1.75; *p*<0.05) (Fig 9B). Post-hoc tests revealed that S_1_ showed significantly higher CS than S_2_ and S_3_ throughout the driving session (*p*<0.05). When road segments were compared, S_1_ showed lower CS in Ci_4_ than in the preceding city segments (Ci_1_, Ci_2_ and Ci_3_; *p*<0.05). For S_3_, CS was lower in the first segments (Hi_1_, Co_1_ and Mo_1_) than, respectively, in the last highway (Hi_5_; *p*<0.001), last country (Co_3_; *p*<0.001) and last mountain segments (Mo_3_, *p*<0.05).

#### Areas of backrest

For the backrest, an AREAxSEGMENT interaction effect was observed for CP (*F*_(42,1680)_ = 6;36; *p*<0.001). Post-hoc tests indicated that whatever the seat, the Right Upperback (RUb) showed lower CP than the other areas of the backrest (LUb, RLb & LLb; *p*<0.01) (Fig 7). Comparing road segments, post-hoc tests showed that CP was higher in Co_3_ than in the preceding country road segments (*p*<0.01). Moreover, Mo_3_ also showed higher CP than the preceding mountain segments for LUb, RUb and RLb (*p*<0.01). In the last two highway segments (Hi_4_ and Hi_5_), CP for RUb was higher than in the preceding ones (*p*<0.01). The LUb CP was also higher in Ci_3_ than in Ci_1_ and Ci_2_ (*p*<0.01). In contrast, the RLb CP was higher in Ci_1_ than in Ci_2_ and Ci_4_ (*p*<0.001), and higher in Ci_3_ than in Ci_4_ (*p*<0.05).

An AREAxSEGMENT interaction effect was observed for CS (*F*_(42,1680)_ = 9.31; *p*<0.001) (Fig 8). RUb showed lower CS than other backrest areas (*p*<0.05). In highway segments, CS for RUb increased with time (Hi_1_ < Hi_2_ < Hi_3_ < Hi_4_ < Hi_5_; *p*<0.05). In mountain segments, CS for LUb and RUb increased with time (Mo_1_ < Mo_2_ < Mo_3_; *p*<0.05). In city segments, CS for LUb and RUb were higher in Ci_3_ than in Ci_1_ and Ci_2_ (*p*<0.05). In country segments, CS for LUb and RUb were lower in Co_1_ than in Co_2_ and Co_3_ (*p*<0.05). However, CS for RLb was higher in Ci_1_ than in Ci_2_, Ci_3_ and Ci_4_ (*p*<0.01).

#### Repositioning movements

**Fig 10:**
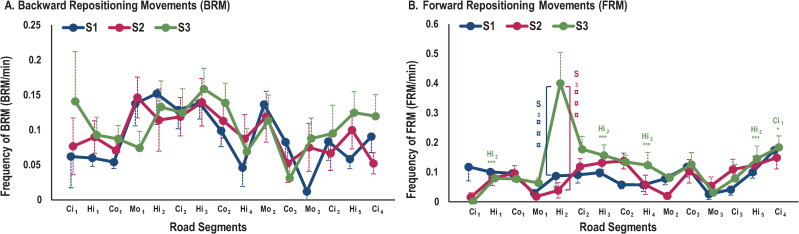
Frequency of RM for each road segment. A) Backward RM. B) Forward RM. * represents significant differences between segments for the same road type (*: p<0.05; **: p<0.01; ***: p<0.001). Colored * represents one seat, black * represents three seats. § represents significant differences between two successive segments (§: p<0.05; §§: p<0.01; §§§: p<0.001). ¤ represents significant differences between seats (¤: p<0.05; ¤¤: p<0.01; ¤¤¤: p<0.001).

Concerning RM frequency, our results indicated an interaction effect from TYPE OF RMxSEGMENTxSEAT (*F*_(28,560)_ = 2.42; *p*<0.001) (Fig 10).

Only a significant difference between seats was observed. S_2_ showed higher frequency of BRM and FRM than S_1_ and S_3_ (*p*<0.001). Moreover, S_3_ frequency of FRM was higher in Ci_4_ than in Ci_1_ (*p*<0.05) and also higher in Hi_2_ than in all other highway segments (*p*<0.001).

### Discomfort

**Fig 11:**
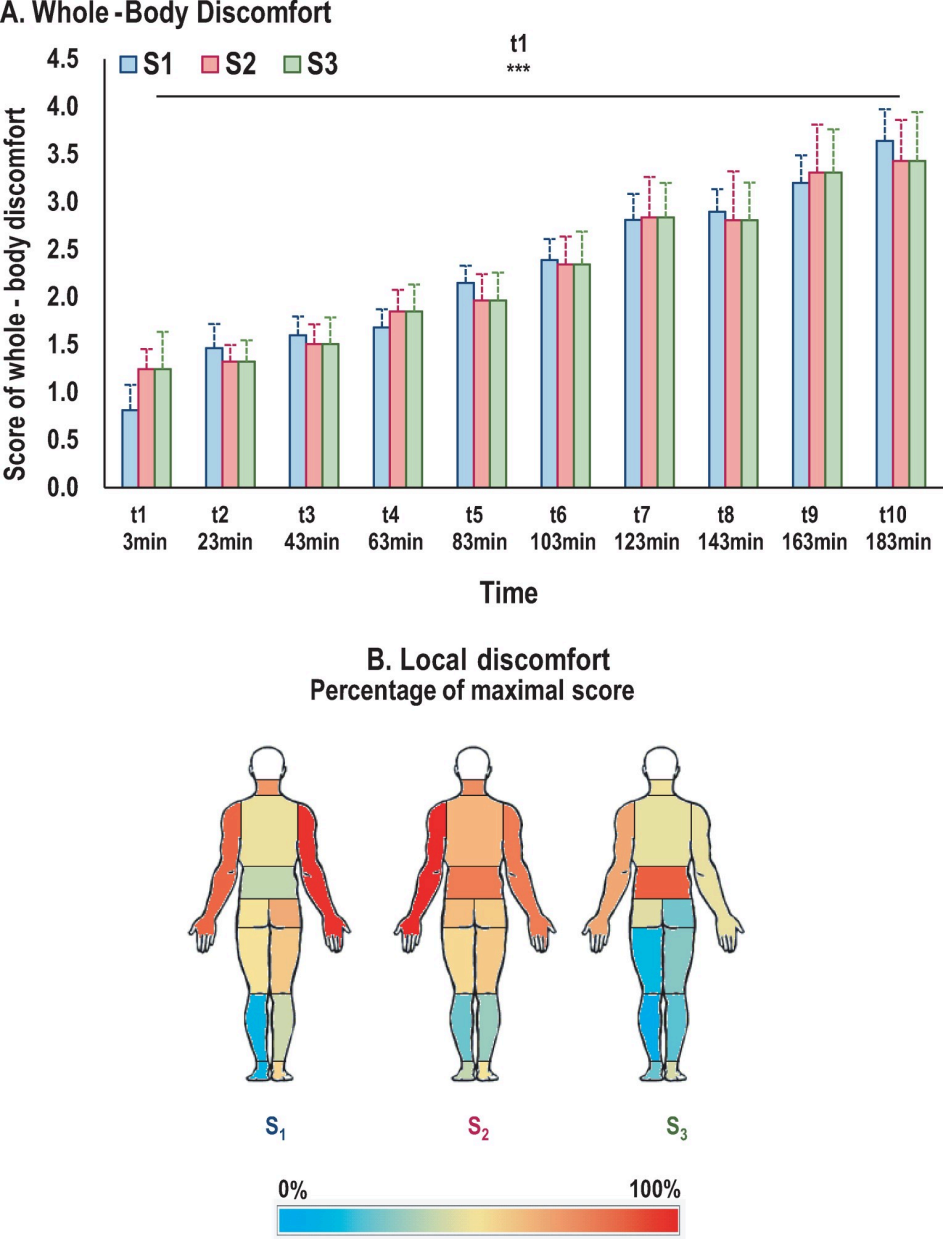
Perceived discomfort. A) Mean scores for whole-body discomfort throughout driving session. Significant increases in discomfort compared to score at t_1_ are represented by ***: p<0.001. B) Visual representation of mean of local discomfort. A color scale shows scores as a percentage of maximum values for each body part.

A main effect of TIME (*F*_(9,369)_ = 69.09; *p*<0.001) was observed on whole-body discomfort scores. Post-hoc tests showed that, for all seats, the discomfort score at the first evaluation (t_start_) was significantly lower than at all subsequent discomfort assessments (from t_23 min_ to t_end_; *p*<0.001) (Fig 11A).

A TIMExBODYPART interaction effect was observed on evolution of discomfort scores for each body part (*F*_(108,4428)_ = 1.64; *p*<0.001). For all body parts and all seats, perceived discomfort increased throughout the driving session. However, the time when this increase began depended on the body part. Post-hoc tests revealed that discomfort appeared earlier for the neck, upperback, arms, and feet, with significant differences from t_start_ discomfort score appearing from t_4_ (63 minutes) to the end of the driving session. Discomfort scores for the buttocks, right thigh, and legs began to differ from the t_start_ score at t_5_ (83 minutes). Finally, the left thigh and lowerback were the last body parts where discomfort was perceived, their score beginning to differ from the t_start_ score at t_6_ (103 minutes). The highest perceived discomfort scores were for both arms, neck, and lowerback (Fig 11B).

## Discussion

The main objective of this study was to define drivers’ sitting behavior and perceptions of discomfort when using different seats under real prolonged driving conditions on a variety of road types. Our results confirm previous findings of an effect from foam hardness on contact pressure and pressure distribution [34, 35]. The firmest of the seats tested here (S_2_) led to higher CP and SPD% than the soft seat (S_1_) and the seat with a suspension system (S_3_). In terms of perceived discomfort, S3 with its suspension system kept local discomfort to a minimum, although all three seats led to a similar general increase in discomfort.

### Analysis of interface pressure between driver and car seat

Interface pressure recorded instantaneously can be used to describe the sitting of the driver with different seats [36, 37]. Pressure distribution, changes in pressure distribution and whole-body vibrations (WBV) are the main factors in sitting discomfort [28]. Using pressure measurements, we found that overall, the driver’s sitting behavior depended on several factors such as seat characteristics, road dynamics, and driving time. Although all three seats’ backrests showed the same CP and CS distribution profiles, with lower values for the right upper back (RUb), their distribution profiles differed for the seat cushion. Softness is known to induce a higher CS and to minimize pressure peaks under ischial tuberosities, including when there are vibrations [35, 38]. In our study, while no differences in cushion CS were observed between the three seats, the S3 distribution profile showed differences between left and right thighs, and between buttocks and bolsters. The CP distribution profiles of S_2_ and S_3_ were similar, with CP highest under the thighs, as already observed by Zenk et al. [39]. However, S_1_ showed uniform CP distribution between buttocks and thighs. S_2_ showed higher CP and higher SPD% on the full cushion mat, which confirmed its firmness. Thus, S_2_ could engender a higher risk of soreness and fatigue, particularly for buttocks and thighs, because of soft tissue compression at these locations [13, 40]. Moreover, Vink & Lips [41] indicated that the contact area at the front of the seat cushion, corresponding to the lower thigh, is more sensitive and could induce additional pain in the legs and feet [28]. The compression caused by the contact constraints of prolonged driving was found to lead to static muscle contractions and a constriction of blood circulation in the lower limbs that can become painful [42]. A prolonged period of driving was reported to require initial adjustments to find a good posture combining physical and environmental constraints [43].

Our results reveal that the cushion CP of S_2_ during the first four segments (Ci_1_, Hi_1_, Co_1_, Mo_1_) differed from that of subsequent segments, becoming stable from the second highway segment (after approximately 1 hour of driving) to the end of the drive. On the other hand, for S_1_ and S_3_, CP increased only in highway segments, where it was significantly lower in Hi1. Concerning CS, the same stabilization was observed after the first two road segments, whatever the seat. These results are consistent with previous studies demonstrating some sitting adjustments during the early phases of the drive [25, 44]. However, these initial adaptations to the seat cushion do not prevent postural fixity for long periods. Maintaining a prolonged driving posture reduces opportunities for movement due to the need to reach commands [12]. Moreover, a stable sitting posture increases contact points and contact surface while decreasing the degree of freedom for movements [10]. Previous studies demonstrated that an increase in repositioning movements (RM) with driving time is clearly associated with the onset of discomfort [19] and used to provide relief from static posture [11]. RM are unconscious, a means to relieve pressure under the soft tissues of the back, buttocks, and thighs, to continue minimizing intervertebral disc loading, and to induce changes in muscle contractions [20]. In our study, backrest pressure data allowed us to identify RM through variations that correspond to a dynamic behaviour, time-dependent and occurring from 30 minutes into the drive [18, 19, 37]. But our findings do not show an effect of the seat, nor of the road segment.

In addition, cushion hardness, composition, and the resulting contact area also have an impact on trunk support. Mergl et al. [45] recommended a firmer cushion to avoid back discomfort even if this meant higher CP under buttocks and thighs. Our findings show that the firmness of S_2_ induced backrest CP similar to that of S_1_ throughout the driving session but applied over a significantly smaller contact area, while S_3_ presented the lowest backrest CP and CS. In a study by Carcone & Keir [46], participants, regardless of their stature, preferred backrests with low contact pressure. Using pressure parameters here helped to highlight the major impact of seat hardness over time, with both sitting behavior and perceived discomfort evolving throughout the driving session.

### Postural discomfort of driver

Driver discomfort is a predictor of the onset of pain in the neck, back, buttocks, and thighs [47]. Mansfield et al. [29] demonstrated that WBV induced higher discomfort ratings than static conditions. Vibrations are transmitted to the driver through the seat, pedals, and steering wheel in real driving conditions [14]. These multiple sources of vibrations, added to postural fixity, foam composition, shape of seat, and individual driver characteristics, are factors that automotive companies need to take into account to optimize the static and dynamic characteristics of the seat [17]. As previously demonstrated [14, 19, 20], the level of whole-body discomfort increased with driving time in our study, whatever the seat. From the second assessment on (t_23min_), discomfort scores became significantly higher than the first scores (t_3min_). Under ideal road conditions and with high-quality vehicle suspension systems, the magnitude of the vibrations perceived through the seat can be low [13]. In our study, road quality was unpredictable, as in real driving conditions, with alternating smooth and rough segments that induced different levels of WBV. S_3_, incorporating a suspension system in the backrest to optimize the dynamic seat characteristics and improve driver comfort, offered better absorption of vibrations in *z*-axis. Unexpectedly, however, S_3_ did not minimize general perceived discomfort, engendering a level of whole-body discomfort similar to S_1_ and S_2_ at the end of the driving task, no doubt due to the muscular and biomechanical consequences of prolonged driving posture. While different types of seats generate different muscular adaptations, drivers may have difficulty differentiating between seats in terms of perceived discomfort. Gyi & Porter [48] reported that a minimum of two hours’ driving is required to differentiate between seats. Lecocq et al. [49] therefore suggested that muscular adaptations linked to seat features are a better way to differentiate between seats than perceived discomfort. Nevertheless, significantly less local discomfort was found with S_3_ here for all body parts except the lower back, no doubt because this prototype was designed for improved suspension and not back support. This seat therefore appears to be more effective in reducing local discomfort than S_1_ and S_2,_ but there is room for improvement to reduce general discomfort and avoid back pain.

### Automotive design implications

Car seat designers are faced with making a compromise between producing seats that delay the onset of static discomfort while avoiding vibration discomfort by minimizing WBV [9]. This balance between static and dynamic factors can be achieved by taking into account the following: drivers’ anthropometry, seat design, and driving environment [1, 8]. Currently, designing cockpits based on driver stature alone is not feasible because it would involve customization to each driver’s characteristics. For example, height, body mass index, hip circumference, and even age or gender all have an impact on sitting posture, notably on pressure parameters [50, 51]. In addition, psychosocial factors like aesthetic or emotional reactions may affect the driver’s perceived comfort [7]. However, manufacturers are trying to prevent the detrimental physical consequences of prolonged posture, like discomfort, fatigue, or pain. Romelfanger & Kolich [52] used big data analytics to underline the importance of considering thigh length in seat design. The hardness of foam was reported to contribute to biceps femoris support and consequently to the loading suffered in the lower back [39]. Lecocq et al. [49] found that firm foam (like that of S_2_) offered better trunk support and delayed fatigue in the back muscles. Neuromuscular fatigue during driving is linked to peripheral and central mechanisms involved in maintaining posture and attention. Therefore, changes in road environment, particularly in visual demands and road curvatures, generate variations in the driver’s attention and may cause central fatigue [53]. Greater stability and better support could be offered by reinforced bolsters [31], which might help to reduce this fatigue. Highways, being straight roads, were found to cause performance deterioration through making lower task demands than curved roads. Conversely, mountain roads led to greater engagement in the task but worse pressure distribution on the seat cushion, with higher SPD% [54]. Moreover, the backrest CP of the soft seats (S_1_ & S_3_) varied only for highway segments, whereas the firm seat (S_2_) fulfilled its support function on all road types, with positioning adjustments only at the beginning of the driving session. Pressure parameters seem appropriate to our approach to testing the driver’s response to the seats on different road types, since they reveal positioning adjustments. This worked particularly well in distinguishing between straight and curved roads, although the distinction between country and city roads was likely complicated by the fact that these road types necessitated more varied lateral and longitudinal accelerations.

Given all the above factors, it might be worth exploring an approach to car seat design that combines softness under the buttocks and thighs to avoid critical pressure points with firmness in the backrest and bolsters to support the trunk and the pelvis. This compromise between reducing contact pressure and providing support might well reduce perceived discomfort.

Seat S_3_ combined these characteristics by offering a soft cushion and low CP and CS on the backrest, doubtless due to its suspension system. Although none of the seats attenuated whole-body discomfort over time, the suspension system had a positive effect on local discomfort by decreasing WBV in *z*-axis. Baucher & Leborgne [10] recommended respecting drivers’ spinal curvatures and suggested that mobility should be encouraged by the seat design. The authors explained that alternating pressure constraints can minimize muscle fatigue and promote fidgeting movements. Fidgeting is recognized as beneficial, helping the driver to feel refreshed by relieving compression that can impede blood flow [19, 20]. Several dynamic solutions have been proposed to make drivers vary their position during driving. For example [11, 55], an integrated seat system with continuous variation of backrest or seat pan angle was shown to reduce perceived discomfort by fostering body movements. This kind of dynamic system, in combination with a seat design incorporating a suspension system for reduced discomfort, may enhance the prolonged driving experience.

### Limitations of the study

The main limitation of this dynamic experiment comes from not recording accelerations. To determine and to highlight the magnitude of WBV, lateral, and longitudinal accelerations, the use of tri-axial accelerometers should be considered. First, this additional device placed on the driver might allow more accurate detection of RM in the three planes of motion [56]. Second, accelerometers placed on the seat and on the car floor could provide objective data to better characterize the different road types. Measuring three-dimensional accelerations from each road segment at different levels (car, seat, and driver) might provide more precise input on the influence of different road types on discomfort. Moreover, this on-board equipment would also allow the speed of the vehicle and its variations to be considered. Kim et al. [57] evaluated the effect of accelerations, decelerations, and curves on shear-forces at the seating interface, using a dummy with several sensors. The authors showed that as vehicle speed increased, so did the shear-forces. Variations in pressure parameters, especially the pressure distribution represented by SPD%, could therefore be partly attributed to variations in velocity and associated shear-forces.

The advantage of our experiment, conducted under real driving conditions, lies in exploiting dynamic conditions representative of the driving environment, rarely explored in the literature. However, by the same token, our experiment is exposed to certain disadvantages since stochastic conditions are more random than those in the laboratory. The differing lengths of time spent in the different road segments, the route-dependent sequencing of segments, and traffic conditions may all interfere with the interpretation of results regarding the four road types. In terms of arm discomfort, moreover, even though the experiment was conducted in real-world conditions, we required drivers to keep their hands on the wheel, which may have affected arm discomfort ratings. Most of the participants are used to driving with a manual transmission and maneuvering alternately with one or two hands, which would explain why this result was obtained for all 3 seats. Finally, the limited sample did not allow us to distinguish anthropometric sub-groups and to evaluate the effect of size or weight. Anthropometry, especially weight and skin surface, are predictors of contact surface [58]. For the above reasons, we believe that future studies taking into account anthropometry, accelerations, and interface parameters would provide even more accurate seat design guidelines for manufacturers [59].

## Conclusion

The aim of this study was to contribute to current recommendations by elucidating the effects of foam hardness, absorption capacities, and road types on drivers’ sitting behavior. We observed a difference in these three seats’ impact on pressure parameters under dynamic conditions. In real driving conditions, there was a difference between firm and soft seats S_1_ and S_2_, with higher CS on the backrest for S_1_, likely due to drivers’ sitting deeper in the seat and leaning more against the backrest. In addition, even though no difference in CP and CS was observed between the three seat cushions, the suspension system in S_3_ reduced local discomfort, especially under buttocks and thighs. However, this seat still needs to be further optimized to mitigate the effect of a prolonged sitting posture, since general discomfort increased with driving time despite the suspension system. Future research should be directed toward supplementary features integrated into the seat to foster movement by drivers. In addition to suspension systems, better support on curved roads, and enhanced pressure distribution under buttocks and thighs could mitigate discomfort from prolonged driving, particularly in the lower back.
